# Editorial on research topic: aldo-keto reductases and role in human disease

**DOI:** 10.3389/fphar.2013.00065

**Published:** 2013-05-21

**Authors:** Yi Jin

**Affiliations:** Department of Pharmacology, University of Pennsylvania Perelman School of MedicinePhiladelphia, PA, USA

Research on Aldo-keto reductases (AKRs) over the years has revealed a remarkable diversity in the reactivity of these soluble NAD(P)(H) oxidoreductases. The AKR enzymes primarily catalyze the reduction of aldehydes and ketones to primary and secondary alcohols, respectively. The 10 known human AKR enzymes can turnover a vast range of endogenous and exogenous substrates, including glucose, steroids, carcinogens, reactive aldehydes, and a variety of carbonyl-containing drugs. These enzymes have been implicated in a number of human diseases, including cancer and disorders of endocrinology and metabolism. AKRs are involved in the development and progression of many cancers, as well as chemotherapeutic drug resistance. AKR1B1 and AKR1B10 are overexpressed in tumors, such as liver, breast, and lung cancer. Several AKRs (AKR1A1, AKR1B10, and AKR1C1-3) are involved in tobacco-carcinogenesis, but they also catalyze the detoxication of nicotine derived nitrosamino ketones. In addition, AKR1C1-3 enzymes play a key role in the regulation of proliferative signaling in hormone dependent cancers. AKR1B1 has long been known for its involvement in diabetic complications. Recent studies now suggest AKR1B enzymes play a key role in inflammatory diseases such as atherosclerosis, sepsis, asthma, uveitis, and colon cancer by mediating oxidative stress-induced inflammatory signals. In recognition of the role of AKR in various diseases, significant efforts have been made in the development of specific/selective AKR1B1 and AKR1C3 inhibitors. The aim of this Research Topic forum is to celebrate the advancements seen in the AKR field via review papers and original articles, as well as to promote future research in understanding the underlying mechanism(s) of the role of AKRs in human disease.

Eight review articles are included in this issue, and are grouped into disease areas (cancer, disorders of endrocrinology and metabolism) after the artile written by Chen and Zhang (2012) which describes the current understanding of the regulation of all human AKR members in relation to disease. The significance of the AKR enzymes for research in various cancers is discussed in four review articles. The article by Matsunaga et al. (2012) focuses on the role AKR1B10 in drug resistance of cancer cells and the development of AKR1B10 inhibitors to reverse chemoresistance. The article by Ruiz et al. (2012) describes the retinaldehyde reductase activity of AKRs in relation to proliferation and tumorigenesis. The paper from the Penning group (Zhang et al., 2012) summarizes the role of AKR in the metabolic activation and detoxication of polycyclic aromatic hydrocarbons and the relevance of phase II conjugation reactions to human lung carcinogenesis. Rižner (2012) provides a review on the role played by AKR1B and AKR1C members in the pathogenesis of endometrial and cervical cancers, as well as other uterine diseases. The aspect that members of AKRs can mediate the timing of parturition through metabolism of progesterone and prostaglandins is discussed by Byrns (2012). The role of AKR1B members in modification and production of signaling molecules is emphasized by Pastel et al. (2012) for a better understanding of the physiological and pathological role of AKR1B enzymes. The article by Tang et al. (2012) focuses on the increases in oxidative stress by AKR1B1 in pathogenesis of diabetic complications such as cardiovascular diseases.

Three Original Research Articles follow the review articles. The article by Fortier and coworkers (Bresson et al., 2012) reports on the prostaglandin *F* synthase activity of AKR1B1 in different models of living cells and tissues. This activity of AKR1B1 represents a novel target to regulate ischemic and inflammatory responses associated with several human pathologic conditions. The article by Laffin and Petrash (2012) examines the expression of AKR1B1 and AKR1B10 across all major human cancer types. Their findings point to the great potential of AKR1B1 inhibition as novel cancer therapeutics. Schulze et al. (2012) describe their studies on basal and regulatory promoter of *AKR1C3* in relation to prostate cancer.

As pointed out in the review article by Pastel et al. (2012) there is a “marked contrast between the abundance of enzymatic data and the small number of reports dedicated to functional studies in a physiological context.” It is my hope that with an emphasis on the role of AKRs in human disease the articles included in this issue will benefit a broad audience and the researchers in the rapidly growing AKR filed.
